# An Advanced Adaptive Control of Lower Limb Rehabilitation Robot

**DOI:** 10.3389/frobt.2018.00116

**Published:** 2018-10-08

**Authors:** Yihao Du, Hao Wang, Shi Qiu, Wenxuan Yao, Ping Xie, Xiaoling Chen

**Affiliations:** Key Lab of Measurement Technology and Instrumentation of Hebei Province Institute of Electric Engineering, Yanshan University, Qinhuangdao, China

**Keywords:** lower limb rehabilitation robot, motion analysis, dual closed loop control, advanced variable impedance control, sliding mode iterative learning control

## Abstract

Rehabilitation robots play an important role in the rehabilitation field, and effective human-robot interaction contributes to promoting the development of the rehabilitation robots. Though many studies about the human-robot interaction have been carried out, there are still several limitations in the flexibility and stability of the control system. Therefore, we proposed an advanced adaptive control method for lower limb rehabilitation robot. The method was devised with a dual closed loop control strategy based on the surface electromyography (sEMG) and plantar pressure to improve the robustness of the adaptive control for the rehabilitation robots. First, in the outer loop control, an advanced variable impedance controller based on the sEMG and plantar pressure was designed to correct robot's reference trajectory. Then, in the inner loop control, a sliding mode iterative learning controller (SMILC) based on the variable boundary saturation function was designed to achieve the tracking of the reference trajectory. The experiment results showed that, in the designed dual closed loop control strategy, a variable impedance controller can effectively reduce trajectory tracking errors and adaptively modify the reference trajectory synchronizing with the motion intention of patients; the designed sliding mode iterative learning controller can effectively reduce chattering in sliding mode control and excellently achieve the tracking of rehabilitation robot's reference trajectory. This study can improve the performance of the human-robot interaction of the rehabilitation robot system, and expand the application to the rehabilitation field.

## Introduction

Recently, the rehabilitation robots have shown great advantages and have attracted more attention in rehabilitation field, which can assist patients in rehabilitation training and effectively alleviate the work pressure of the therapist (Lo et al., 2010). Currently, according to the training mode in the rehabilitation process, the rehabilitation robots are mainly divided into two types: passive training and active training. The former has been widely applied in clinic, and has brought some effects for patients, but it lacks active participation of patients and may leads to unreasonable and insufficient recovery. The latter can provide appropriate assistance according to patients' active motion intention and state, which contributes to the recovery of motor nerves and accelerate the rebuilding of motor function. Evidence-based medicine also shows that active rehabilitation training has better recovery effects on patients (Costandi, 2014). In the active rehabilitation training process, the control strategy can be adjusted adaptively according to motion state of the patient. Many studies on this have been done as following:

In order to realize the active control of rehabilitation robot, effective motion intention recognition and motion state analysis is very important. Surface electromyography (sEMG), as an information which can reflect the muscle status (Wu et al., 2010), has been used in motion intention recognition (Amsüss et al., 2014; He et al., 2015) and interaction control of human-robot system (Meng et al., 2014). Human motion intention recognition methods are mainly divided into discrete action classification and continuous motion analysis (Kawase et al., 2014; Hou et al., 2016). The discrete action classification method can be used in the rehabilitation robot control system of early rehabilitation training for patients, but the human-robot interaction level is low, while the continuous motion analysis method can be used in adjusting the rehabilitation robots' degree of assistance in real-time according to the patients' motion intention and motion ability. For example, the skeletal muscle model is used to predict the multi-joint angle, but it is not suitable for interaction control of the human-robot system since the model has many unknown parameters and low accuracy (Buchanan et al., 2004; Meng et al., 2015). The musculoskeletal model is simplified in some researches, for example, joint-angle model was established by introducing the muscle activity and time domain features (Koo and Mak, 2005); the k-order dynamic model was designed by using the LS-SVR method to predict the joint angle (Tang et al., 2016). By establishing the regression model between sEMG and joint angles, the prediction accuracy is significantly improved, but the modeling takes long time, which may cause patient muscle tired. Relevant studies have shown that the prediction errors may significantly increase under the condition of muscle fatigue, and it is difficult to guarantee the interaction control security of the human-robot system (Li Z. et al., 2015). In addition, in some studies, the sEMG signal was applied to predict the muscle strength of the limb in order to realize the active control of the rehabilitation robot (Duschau-Wicke et al., 2010), but the prediction accuracy of muscle strength still need to be improved. Therefore, it is necessary to comprehensively consider the sEMG, joint angle and human-robot interaction force to realize an accurate motion state analysis.

Furthermore, many studies concentrates on how to design the adaptive control strategy of rehabilitation robot in the active training process. The impedance control method, as a commonly intelligent control method for rehabilitation robot, have been introduced into rehabilitation robot control (Jezernik et al., 2004; Xie et al., 2016), which can improve the interaction performance of the human-robot system by adjust the assistance level according to patients' motion intention and motion state. However, there are some limitations on the traditional constant coefficient impedance control method because the parameters of the human-robot system are preset and cannot be adjusted according to the changes of the patient's motion state in real time. Therefore, the variable impedance control method was proposed to adjust the gait training speed within the virtual channel according to the plantar pressure (Kiguchi and Hayashi, 2012), but the virtual channel varies from person to person. Because the sEMG signals can describe the motion state and reflect changes of human damping and stiffness in the human-robot system (Rahman et al., 2014), it has been introduced to the impedance control model in some researches. For example, muscle activity information has been used in the rehabilitation robot control system to adjust the control speed (Rahman et al., 2013), but the performance of control method still need to be improved when patient's motion intention and human-robot interaction force are variable. Therefore, it will be helpful to improve the adaptive ability of rehabilitation robot control if the impedance parameters of the control system are adjusted considering the sEMG, joint angle and human-robot interaction force together.

In this paper, we proposed an advanced adaptive control method for lower limb rehabilitation robot, which was designed with a dual closed loop control strategy based on the sEMG and plantar pressure. Firstly, we carried out motion analysis of human lower limbs with least squares extreme learning machine (LS-ELM) algorithm to obtain the desired trajectory of patients. Then, the designed variable impedance control was used to adaptively correct the desired trajectory according to patients' active motion intention and obtained the reference trajectory of the rehabilitation robot. Finally, the designed SMILC was used to track the reference trajectory and realize the adaptive control of rehabilitation robot, which can enhance the compliance and the robustness of the lower limb rehabilitation robot control system in training. This study can effectively improve the performance of the human-robot interaction and the robustness of control in the rehabilitation robot system.

## Human-robot system modeling

To verify the adaptive control method of the lower limb rehabilitation robot proposed in this study, we first established a human-robot system model as the control object for further study.

**Figure 1:**
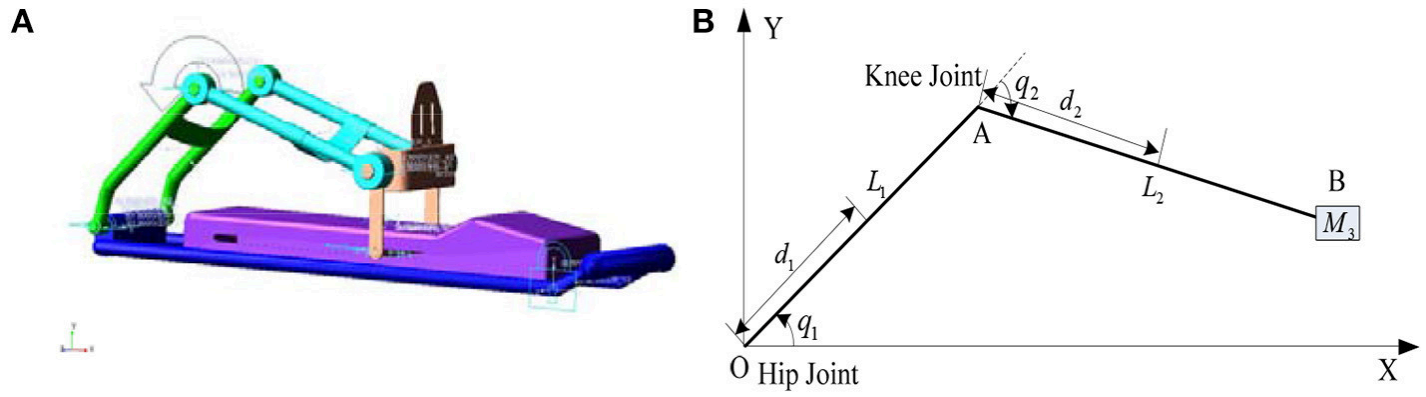
Lower limb rehabilitation robot. **(A)** Lower limb rehabilitation robot model. **(B)** Diagram of lower limb rehabilitation robot.

In this study, we chose the lower limb rehabilitation robot with one degree of freedom as the object, which could complete the horizontal extension and flexion movement through the rod and the pedal, Figure 1 showed the model and simplified diagram of the lower limb rehabilitation. In order to reduce the modeling complexity, the rehabilitation robot and human lower limb are considered to be a single unit and simplified as a two-link series mechanism.

The Cartesian coordinate system is established with the hip joint as the origin, as shown in (Figure 1B). The coordinate of robot's end point B is calculated through kinematics:

(1) $$\begin{array}{l} \text{X}=\left[\begin{array}{c} L_{1}\cos q_{1}+L_{2}\cos\left(q_{1}+q_{2}\right)\\ L_{1}\sin q_{1}+L_{2}\sin\left(q_{1}+q_{2}\right) \end{array}\right] \end{array}$$

where *L*_*i*_ is the length and *q*_*i*_ is the deflection angle of the *i*-th bar. The deflection angle of the joint can be solved through inverse kinematics:

(2) $$\begin{array}{l} \text{q}=\left[\begin{array}{c} \arcsin\left(\frac{-L_{2}s_{2}}{\sqrt{x_{B}^{2}+y_{B}^{2}}}\right)+\arctan\left(\frac{y_{B}}{x_{B}}\right)\\ \text{arcos}\left(\frac{L_{1}^{2}+L_{2}^{2}-x_{B}^{2}-y_{B}^{2}}{2L_{1}L_{2}}\right) \end{array}\right] \end{array}$$

Considering the influence of human movement on the human-robot system, the mapping torque of the human active power in robot space is used as part of the drive torque of the human-robot system, and the dynamic model of the human-robot system can be described as:

(3) $$\begin{array}{l} \tau_{r}+\tau_{hr}^{h}=M(q)\ddot{q}+H(q,\dot{q})+G(q) \end{array}$$

where $\text{q}={\left[q_{1}\;q_{2}\right]}^{T}$ is the angle of hip joint and knee joint, ***M***(*q*) is the positive definite inertia matrix of the human-robot system, $H(q,\dot{q})$ is the Coriolis force and the centrifugal correlation matrix, **G**(***q***) is the gravity matrix, $\tau_{hr}^{h}$ is the equivalent torque of human active moment in robot space, and **τ**_***r***_ is the driving torque provided by the robot.

The human active force and gravity are both considered in the process of human-robot system modeling, which can improve the accuracy of the human-robot system modeling and interaction performance for the human-robot system. In this paper, a dual closed loop control strategy based on the sEMG signals and plantar pressure was proposed to realize the adaptive control of the human-robot system.

## Adaptive control of human-robot system

### Control strategy of human-robot system

**Figure 2:**
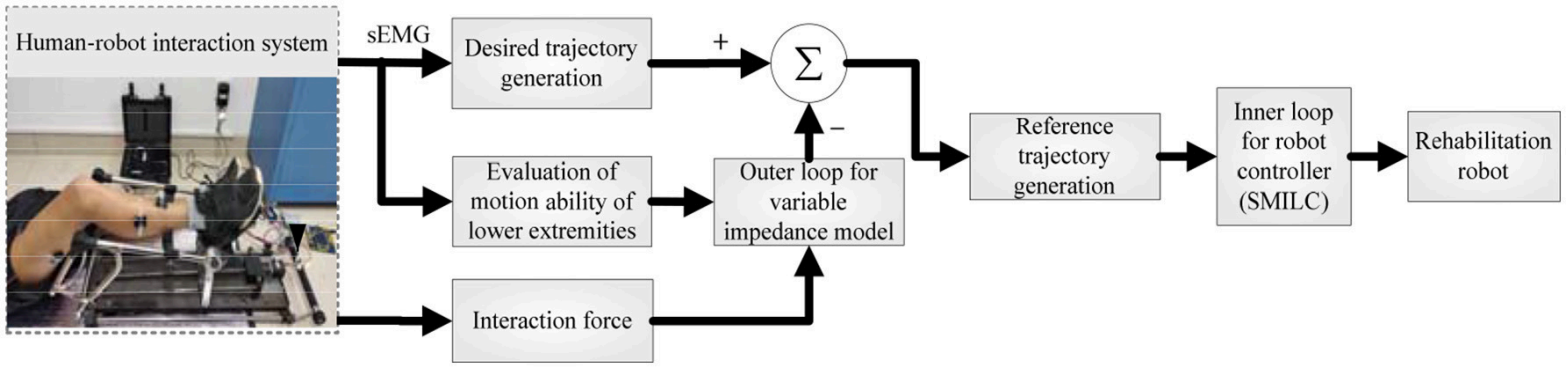
Adaptive control principle of the human-robot system.

To improve the performance of human-robot interaction and compliance control of rehabilitation robot, a dual closed loop control strategy based on sEMG signals and the human-robot interaction force (plantar pressure) is designed for the human-robot system, which is consist of the variable impedance control in the outer loop and the position control in the inner loop. The variable impedance control model based on sEMG and the plantar pressure is designed to obtain the reference trajectory that reflects the patient's motion intention and motion ability by correcting the patient's desired trajectory. Then, the sliding mode iterative learning control algorithm based on a variable boundary saturation function is designed to track the reference trajectory, which performs steady trajectory tracking and improves the robustness of the control system, as shown in Figure 2.

### Desired trajectory generation based on human motion intention

**Figure 3:**
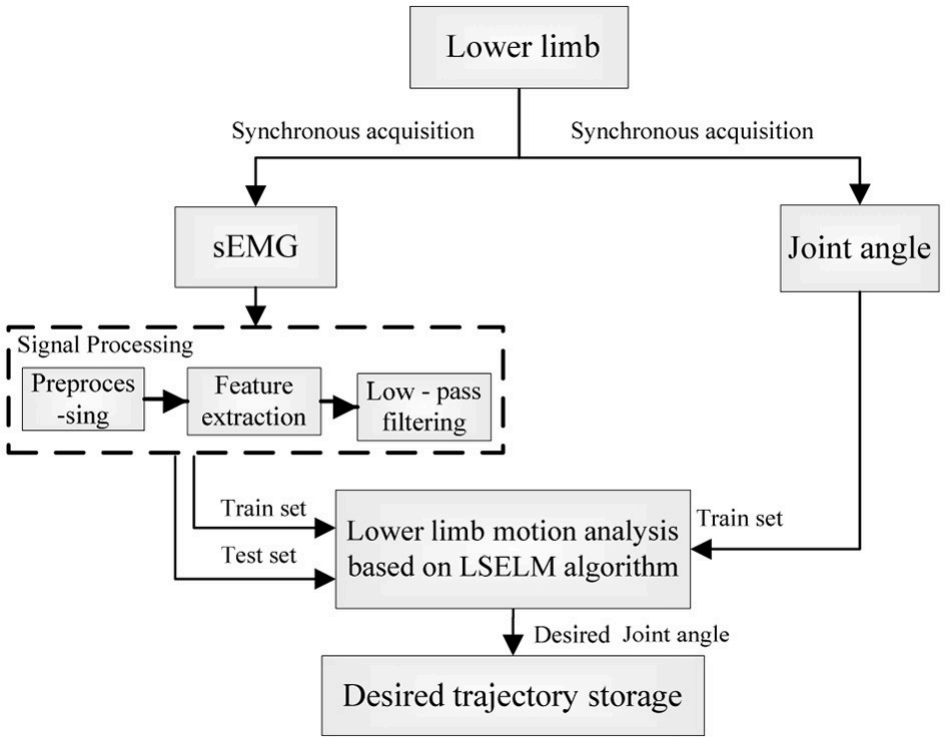
Principle of the desired trajectory generation of the human-robot system.

To obtain the desired trajectory of rehabilitation robot synchronizing with the human motion intention, we established a nonlinear motion analysis model between sEMG and the joint angle. In order to ensure the real-time performance, the desired trajectory was generated by using the least squares extreme learning machine (LS-ELM) algorithm (Li Q. L. et al., 2015), as shown in Figure 3.

The WL (Wave Length) is extracted as the sEMG feature:

(4) $$\begin{array}{l} WL=\overset{N-1}{\underset{i=1}{\sum }}|\xi_{i+1}-\xi_{i}| \end{array}$$

where ξ_*i*_ is the pretreated sEMG signal and *N* is the number of sampling point over a period. The signals were filtered with a 1 Hz low-pass Butterworth filter, and then were normalized.

Taking the lower limb hip joint angle as an example. The inputs of LS-ELM network are the sEMG features *x*_*j*_ of the tibialis anterior muscle and vastus rectus muscle, and the outputs are the hip joint angle θ_*h*_◦

(5) $$\begin{array}{l} \{\begin{array}{l} \theta_{h}=\left[\theta_{1},\cdots ,\theta_{i},\cdots ,\theta_{n}\right]\\ x_{j}=\left[x_{j,1},\cdots ,x_{j,i},\cdots ,x_{j,n}\right],j=1,\cdots ,k \end{array} \end{array}$$

where *n* is the number of training sample and the *k* is the number of input channels.

The hidden layer excitation function is sigmode function:

(6) $$\begin{array}{l} G(z)=\frac{1}{1+e^{-z}} \end{array}$$

The desired output model is:

(7) $$\begin{array}{l} \theta_{h}=\overset{L}{\underset{i=1}{\sum }}\beta_{i}G_{i}\left(\alpha_{i}\times x_{i}+b_{i}\right) \end{array}$$

where *L* is the number of hidden layer nodes, $a_{i}={\left[\alpha_{i1},\alpha_{i2},\cdots \;,\alpha_{in}\right]}^{T}$ is the weight between the *i*-th hidden layer node and input node, *b*_*i*_ is the threshold of the *i*-th hidden layer node, and $\beta_{i}={\left[\beta_{i1},\beta_{i2},\cdots \;,\beta_{iL}\right]}^{T}$ is the connection weight between the output layer node and *i*-th hidden layer node. Deforming the formula (7) with the existing methods (Huynh et al., 2008; Xie et al., 2016; Du et al., 2017; Li et al., 2017) as:

(8) $$\begin{array}{l} \theta_{h}=\left(x\cdot a\right)\cdot \beta \end{array}$$

According to the generalized inverse matrix theory of Moore-Penrose: $x\times \alpha =\theta_{h}{\theta_{h}}^{+}G^{-1}\left(\theta_{h}\beta^{+}\right)$, set that $Z={\theta_{h}}^{+}G^{-1}\left(\theta_{h}\beta^{+}\right)$, and we can obtain that:

(9) $$\begin{array}{l} x\cdot \alpha =\theta_{h}Z \end{array}$$

According to the least squares principle, when ***Z*** is randomly generated, the input weight α, offset ***b*** and output weight β are obtained.

We conducted lower limb motion analysis by using the LS-ELM (Least squares extreme learning machine) algorithm, and obtained the desired trajectory of rehabilitation robot synchronizing with the patient's motion intention. The desired trajectory was then used in the variable impedance control of human-robot system to generate the reference trajectory.

### Adaptive compliance control of the human-robot system

**Figure 4:**
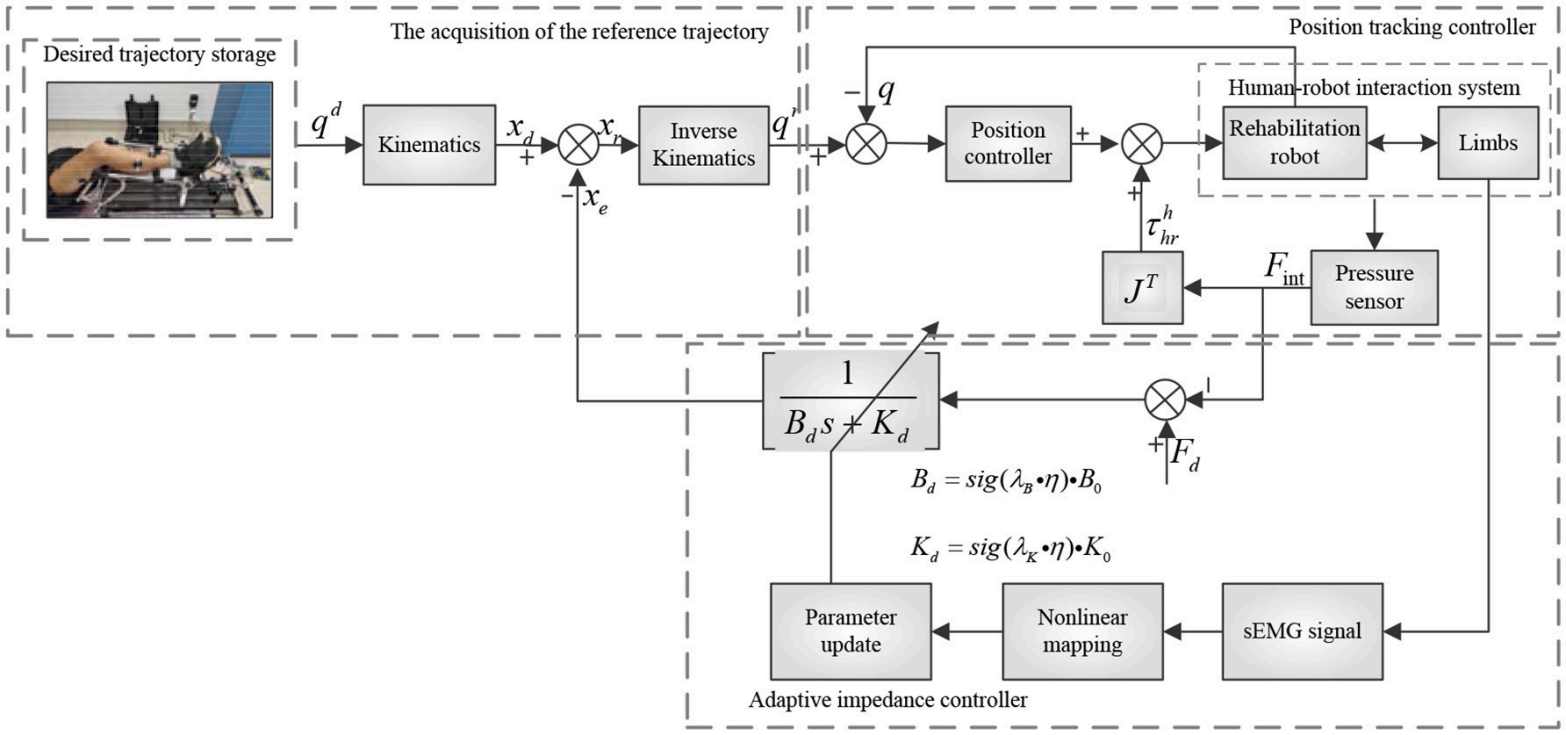
Principle of variable impedance control.

To realize human-robot interaction and compliance control of rehabilitation robot control system, we proposed an advanced adaptive control method for lower limb rehabilitation robot. The method was a dual closed loop structure with variable impedance control in the outer loop and position control based on SMILC in the inner loop, as shown in Figure 4. In the outer loop, the variable impedance controller was designed with impedance coefficients corrected in real-time by the lower limb sEMG activity and muscle contribution rate, which can realize the adaptive adjustment of reference trajectory of the robot according to human stiffness and damping. In other words, the desired rehabilitation robot trajectory was corrected by the lower limb sEMG and human-robot interaction force, and the reference trajectory was obtained synchronizing with patient's motion intention and ability. In the inner loop, a sliding mode iterative learning control algorithm based on variable boundary saturation function is designed for position controller to realize the tracking of reference trajectory. The design of the algorithm could reduce the sliding mode chattering effectively and improve the robustness of the control system.

#### The variable impedance control

The impedance control is a second order model that can denote the ideal dynamic relationship between the robot terminal position and human-robot interaction force. In other words, the desired trajectory of rehabilitation robot is adjusted according to the changes of the plantar pressure, and the reference trajectory is generated according to patients' motion ability. The specific model is designed as follows:

(10) $$\begin{array}{l} {\text{F}}_{\text{int}}-{\text{F}}_{d}={\text{M}}_{d}\left({ẍ}_{d}-{ẍ}_{r}\right)+{\text{B}}_{d}\left({ẋ}_{d}-{ẋ}_{r}\right)+{\text{K}}_{d}\left({\text{x}}_{d}-{\text{x}}_{r}\right) \end{array}$$

(11) $$\begin{array}{l} \tau_{hr}^{h}={\text{J}}^{T}{\text{F}}_{\text{int}} \end{array}$$

where **M**_*d*_, **B**_*d*_, and **K**_*d*_ are the inertia matrix, damping matrix and stiffness matrix respectively; ***x***_*d*_ and ***x***_*r*_ are the terminal position desired trajectory and reference trajectory of the rehabilitation robot respectively; ***J*** is the Jacobian matrix; **F**_*d*_ is the ideal static balance force of human-robot; **F**_int_ is human-robot interaction force.

Since the lower limb active force of patient was small, the effect of acceleration was neglected, and by only considering the damping and stiffness coefficients, we could get that:

(12) $$\begin{array}{l} {\text{F}}_{d}-{\text{F}}_{int}={\text{B}}_{d}\left({ẋ}_{d}-{ẋ}_{r}\right)+{\text{K}}_{d}\left({\text{x}}_{d}-{\text{x}}_{r}\right) \end{array}$$

Formula (13) was obtained by the *s* transforming:

(13) $$\begin{array}{l} {\text{x}}_{e}=\frac{{\text{F}}_{d}-{\text{F}}_{\mathrm{int}}}{{\text{B}}_{d}\cdot s+{\text{K}}_{d}} \end{array}$$

where ***x***_*e*_ = ***x***_*d*_ − ***x***_*r*_ is the desired trajectory correction of the rehabilitation robot. Therefore, the reference trajectory in joint space ***x***_*r*_ = ***x***_*d*_ − ***x***_*e*_ was obtained by inverse kinematics as ***q***^*r*^.

In rehabilitation training, the damping and stiffness of the lower limb changes with human active movement, showing that the change of muscle activity makes the traditional impedance control model unable to meet the requirement of the active compliance control of human-robot system. Therefore, muscle activity was introduced to establish the nonlinear mapping function and adjust the impedance parameters according to human motion (Lloyd and Besier, 2003), making the reference trajectory of rehabilitation robot more in line with the patient's movement ability.

The muscle activity is expressed as:

(14) $$\begin{array}{l} a_{j}=\frac{{e^{A_{j}}}^{{\text{u}}_{j}\left(t\right)}-1}{e^{A_{j}}-1} \end{array}$$

where ***u***_*j*_(*t*) is the sEMG signals after preprocessing and normalization, and *A*_*j*_ is the nonlinear coefficient of the model between sEMG and muscle activity, whose scope is −3 ~ 0.

Lower limb activity η is defined as:

(15) $$\begin{array}{l} \eta =\overset{N}{\underset{j=1}{\sum }}\omega_{j}\cdot a_{j} \end{array}$$

(16) $$\begin{array}{l} \omega_{j}=\frac{RMS_{i}\left(j\right)}{\overset{N}{\underset{j=1}{\sum }}RMS_{i}\left(j\right)} \end{array}$$

where ω_*j*_ is the contribution rate of the *j*-th muscle, and *RMS*_*i*_(*j*) is the mean square root of the sEMG signals.

The damping and stiffness coefficients of the impedance equation can be adjusted:

(17) $$\begin{array}{l} B_{d}=sig\left(\lambda_{B}\cdot \eta \right)\cdot B_{0} \end{array}$$

(18) $$\begin{array}{l} K_{d}=sig\left(\lambda_{K}\cdot \eta \right)\cdot K_{0} \end{array}$$

where λ_*B*_ and λ_*K*_ are the damping coefficient and stiffness gain coefficient respectively; *B*_0_ and *K*_0_ are the initial impedance coefficients; *B*_*d*_ and *K*_*d*_ are the modified impedance coefficients; and *sig*(^*^) is the sigmoid function that limits *B*_*d*_ and *K*_*d*_ in the scope of $\frac{B_{0}}{2}\sim B_{0}$ and $\frac{K_{0}}{2}\sim K_{0}$.

The variable impedance control model, established based on human lower limb sEMG, can adaptively adjust the impedance parameter according to the changes of lower limb activity, and correct the desired trajectory of the rehabilitation robot and generate a reference trajectory, which is in greater agreement with patients' motion ability. Then, adaptive control of the human-robot system is performed according to reference trajectory tracking.

#### The position control based on SMILC

**Figure 5:**
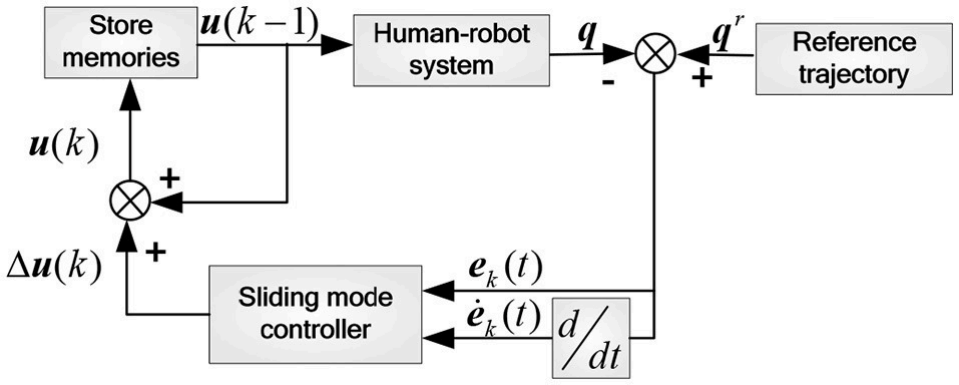
Principle of inner loop position control.

Involuntary tremble of lower limb and periodic interference caused by repetitive training may induce some unknown uncertainties in the human-robot system model, which affect the accuracy and stability of reference trajectory tracking of the rehabilitation robot. Therefore, the sliding mode iterative learning control algorithm based on the variable boundary saturation function is proposed in position control. This algorithm combines iterative learning control and sliding mode variable structure control to suppress the inhibitory periodic and non-periodic disturbances, and replaces the symbol function in iterative learning control algorithm with a variable boundary saturation function to improve the performance of rapidity and robustness of the control system, as shown in Figure 5.

Considering factors such as the modeling errors and parameters variation of the human-robot system, the dynamic model is corrected as:

(19) $$\begin{array}{l} M(q)\ddot{q}+N(q,\dot{q})=u+\tau_{hr}^{h}+\tau_{d} \end{array}$$

where $N(q,\dot{q})=H(q,\dot{q})+G(q)$, **u** is the robot control torque, and **τ**_*d*_ is the repetitive and non-repetitive disturbance caused by rehabilitation robot vibration and human tremble.

The overall control law of the *k*-th iteration is:

(20) $$\begin{array}{l} u\left(k\right)=u\left(k-1\right)+\Delta u\left(k\right) \end{array}$$

where Δ*u*(*k*) is the sliding mode controller output in the *k*-th iteration, *u*(*k*−1) is the control variable of the (*k*-1)-th iteration, and *u*(*k*) will be stored in memory as the input for the next iteration.

The *k*-th error and error ratio of the control system are set as:

(21) $$\begin{array}{l} \text{e}={\left[\begin{array}{cc} q_{1}^{r}-q_{1} & q_{2}^{r}-q_{2} \end{array}\right]}^{T}={\left[\begin{array}{cc} e_{1} & e_{2} \end{array}\right]}^{T} \end{array}$$

(22) $$\begin{array}{l} ė\left(t\right)=\frac{e\left(t\right)-e\left(t-1\right)}{\Delta t} \end{array}$$

where Δ*t* is the time interval between two sampling points, and the sliding mode function is designed as:

(23) $$\begin{array}{l} \text{s}=\text{C}\text{e}+ė=\left[\begin{array}{c} c_{1}e_{1}+{ė}_{1}\\ c_{2}e_{2}+{ė}_{2} \end{array}\right] \end{array}$$

where *c*_1_ and *c*_2_ are the sliding mode coefficients.

(24) $$\begin{array}{l} \dot{s}=\left[\begin{array}{c} c_{1}{\dot{e}}_{1}+{\ddot{e}}_{1}\\ c_{2}{\dot{e}}_{2}+{\ddot{e}}_{2} \end{array}\right]=\left[\begin{array}{l} c_{1}{\dot{e}}_{1}\\ c_{2}{\dot{e}}_{2} \end{array}\right]+\left[\begin{array}{c} {\ddot{q}}_{1}^{r}\\ {\ddot{q}}_{2}^{r} \end{array}\right]\\ \;-M^{-1}(u+\tau_{hr}^{h}+\tau_{d}-N) \end{array}$$

To reduce the chattering in sliding mode control, saturation function based on nonlinear feedback is used to replace the function based on linear feedback in the boundary layer, which can enable the system state to reach the sliding surface in limited time and improve the system robustness. Therefore, we defined the exponential approach law with the saturation function based on the nonlinear feedback:

(25) $$\begin{array}{l} ṡ=-\varepsilon \text{sat}\left(s\right)-ks=\left[\begin{array}{c} -\varepsilon_{1}\text{sat}\left(s_{1}\right)-ks_{1}\\ -\varepsilon_{2}\text{sat}\left(s_{2}\right)-ks_{2} \end{array}\right] \end{array}$$

where **ε**_1_ and **ε**_2_ are strictly positive real numbers.

(26) $$\begin{array}{l} \text{sat}(s)=\{\begin{array}{cc} \text{sgn}(s) & \left|s\right|>\Phi \\ {\left(\frac{s}{\Phi (s)}\right)}^{\alpha } & \left|s\right|\leq \Phi \end{array} \end{array}$$

where Φ is the boundary layer thickness, Φ > 0, $0<\alpha =\frac{p}{q}<1$, *p* and *q* are positive odd numbers. We combined formula (24) with (25) and designed the control law:

(27) $$\begin{array}{l} \text{u}=\text{M}\left(\left[\begin{array}{c} c_{1}{ė}_{1}\\ c_{2}{ė}_{2} \end{array}\right]+\left[\begin{array}{c} {\ddot{q}}_{1}^{d}\\ {\ddot{q}}_{2}^{d} \end{array}\right]+\varepsilon sat\left(s\right)+ks\right)+\text{N}-\tau_{hr}^{h}-\tau_{d} \end{array}$$

Setting **τ**_*dc*_ as the estimated value to replace **τ**_*d*_, whose upper and lower bounds to **τ**_*U*_ and **τ**_*L*_, and then put them into the formula (25), we could get:

(28) $$\begin{array}{l} ṡ=-\varepsilon \cdot \text{sat}\left(\text{s}\right)-k\text{s}-\left({\bar{\tau }}_{\text{d}}-{\bar{\tau }}_{\text{d}\text{c}}\right) \end{array}$$

(29) $$\begin{array}{l} s\dot{s}=-\varepsilon s\;\cdot \text{ sat}(s)-ks-s({\bar{\tau }}_{d}-{\bar{\tau }}_{dc}) \end{array}$$

where ${\bar{\tau }}_{d}={\text{M}}^{-1}\tau_{d}$ and ${\bar{\tau }}_{dc}={\text{M}}^{-1}\tau_{dc}$. The Lyapunov function was set:

(30) $$\begin{array}{l} V=\frac{1}{2}{\text{s}}^{2} \end{array}$$

For the stabilization of sliding mode control system, $\underset{t\to 0}{\lim}s\dot{s}<0$, that is:

(31) $$\begin{array}{l} {\bar{\tau }}_{dc}=\{\begin{array}{c} {\bar{\tau }}_{L},s>0\\ {\bar{\tau }}_{U},s<0 \end{array} \end{array}$$

Setting ${\bar{\tau }}_{m}=\frac{{\bar{\tau }}_{U}-{\bar{\tau }}_{L}}{2}$, ${\bar{\tau }}_{p}=\frac{{\bar{\tau }}_{U}+{\bar{\tau }}_{L}}{2}$, and the sliding mode control law is that:

(32) $$\begin{array}{l} u\left(k\right)=u\left(k-1\right)+\text{M}\left(\left[\begin{array}{c} c_{1}{ė}_{1}\\ c_{2}{ė}_{2} \end{array}\right]+\left[\begin{array}{c} {\ddot{q}}_{1}^{r}\\ {\ddot{q}}_{2}^{r} \end{array}\right]+\varepsilon sat\left(s\right)+ks\right)\\ +\text{N}-\tau_{hr}^{h}-\text{M}\left({\bar{\tau }}_{p}-{\bar{\tau }}_{m}\text{sgn}\left(s\right)\right) \end{array}$$

The sliding mode iterative learning control algorithm, based on the variable boundary saturation function, was used in tracking the reference trajectory of the rehabilitation robot. By sensing the human-robot interaction force and suppressing periodic and non-periodic disturbances, we can quickly complete the tracking of reference trajectory and improve system control robustness, realizing adaptive compliance control of the human-robot system.

## Results and discussion

### Subjects

Seven healthy subjects (aged 25 ± 2 years old) without any previous history of neural or physiological disorders participated in this experiment. Before the experiments, each subject provided informed consent and was informed of the experimental requirements. The experiment was approved by the ethical review board of Yanshan University. To avoid the influence of fatigue, all subjects were in a good state of mind and had not undergone strenuous exercise with lower limb recently.

### Experimental protocol

**Figure 6:**
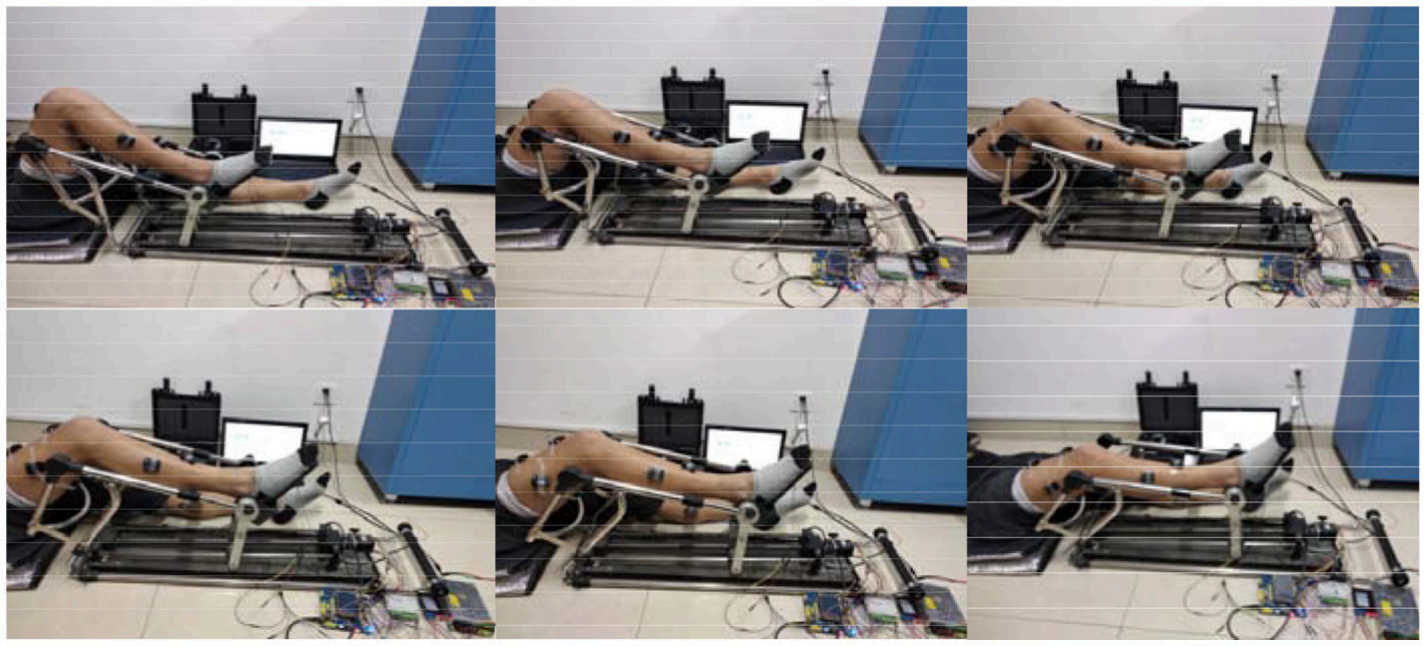
Experimental paradigm and collection points.

In order to verify the effectiveness of the proposed method, the horizontal extension and flexion movement of the lower limb was chosen as the experimental paradigm. And seven healthy subjects (S1~S7, five males, two females, 25 ± 2 years old) were selected for analysis to avoid secondary injuries in patients by accident. The extension period was set to 5 s, and the American Delsys company Trigno™ Wireless EMG system was used to synchronously capture the subject's right leg muscle sEMG signals and joint angles, as shown in Figure 6. We chose the Vastus Rectus Muscle (VR), Vastus Lateralis Muscle (VL), Vastus Medialis Muscle (VM), Semitendinosus Muscle (SM), Biceps Muscle (BM), and Tibialis Anterior Muscle (TA) as data collection points. The researched method was conducted to analyze the adaptive compliance control of the human-robot system for all the subjects.

### Experimental results

#### The prediction of joint angles based on sEMG

**Figure 7:**
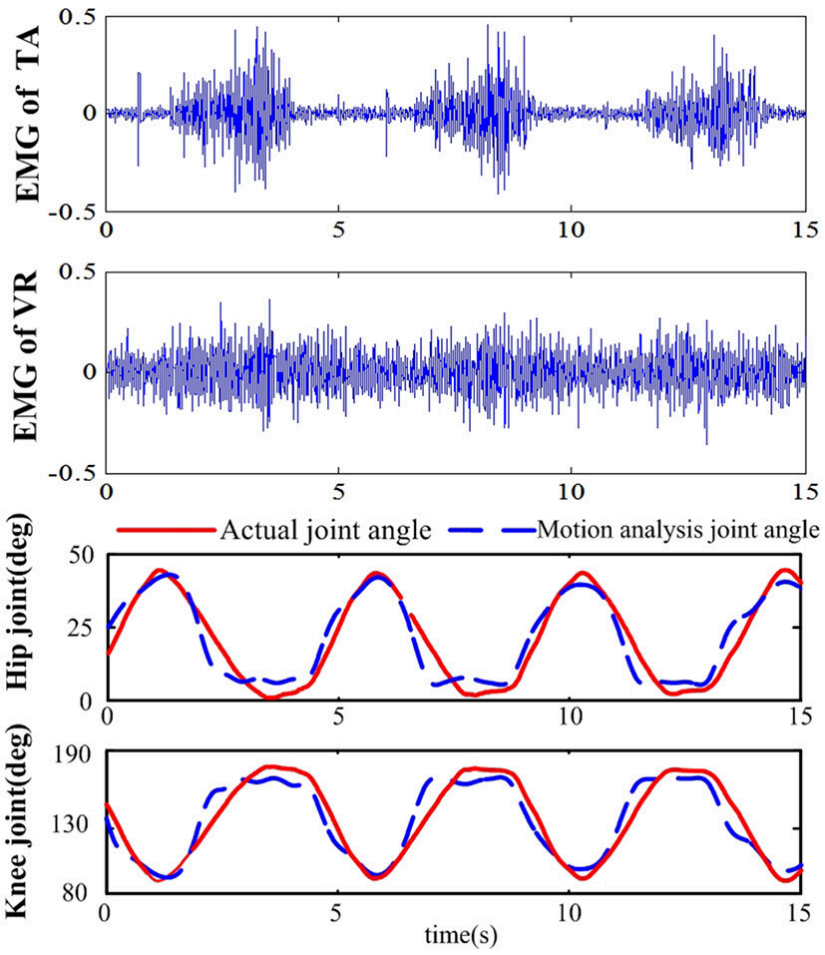
Results of the joint angle and motion analysis based on sEMG.

The joint angle in lower limb extension motion of the 7 subjects was predicted by sEMG signals with the LS-ELM algorithm to realize the continuous motion analysis. The sEMG signal and the predicted joint angle of subject S2 in one training process was shown in Figure 7. The sEMG signals of the VR and TA showed obvious periodicity, and the predicted joint angle were consistent with the actual joint angle. Table 1 shows the results of predicted joint angles of the seven subjects, including the training time, testing time and analysis errors. The average training time of seven subjects' motion is 6.9 ms, the time for motion recognization is 2.9 ms, and the RMSE of hip joint and knee joint angle are respectively 7.55° and 7.26°, which meet the requirement of the desired trajectory generation in real-time and accuracy performance.

**Table 1:** The average time and RMSE results of motion analysis.

**Subjects**	**The average time (s)**		**The RMSE of joint angle (deg)**	
	**Training time**	**Analysis time**	**The RMSE of hip joint angle**	**The RMSE of knee joint angle**
S1	0.0032	0.0022	7.59	8.01
S2	0.0114	0.0067	7.94	8.12
S3	0.0108	0.0039	6.56	6.64
S4	0.0106	0.0031	6.85	6.23
S5	0.0046	0.0015	8.66	8.34
S6	0.0012	0.0005	8.15	8.16
S7	0.0062	0.0026	7.12	7.45
Average	0.0069	0.0029	7.55	7.26

#### The adjustment of lower limb activity and impedance coefficients

**Figure 8:**
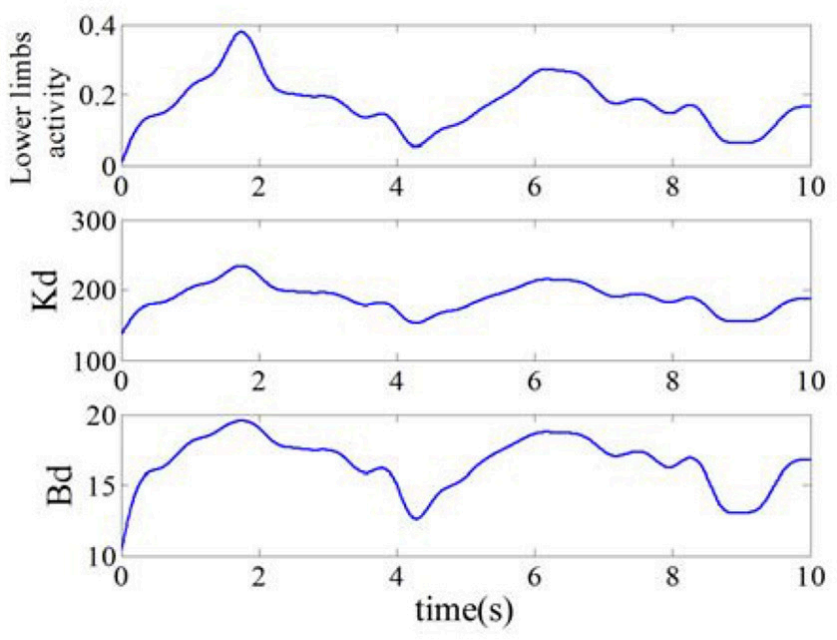
Lower limb activity and impedance coefficients.

The curves of the lower limb activity and impedance coefficients was computed according to formulas (15), (17), and (18) separately, as shown in Figure 8. The curve of lower limb activity indicated the motion state of the subject, and the tendency of the impedance coefficients *B*_*d*_ and *K*_*d*_ were in similar with that of the lower limb activity. For example, the value of lower limb activity decreased in the duration of 1.8~4 s, and the value of the impedance coefficients *B*_*d*_ and *K*_*d*_ decreased also. Therefore, the impedance coefficients can be adjusted according to human motion activity and then can be used to correct the desired trajectory. In this paper, the initial impedance coefficients were set as *B*_0_ = 20 and *K*_0_ = 270, the gain coefficients were λ_*B*_ = 5 and λ_*K*_ = 10, and the impedance coefficients *B*_*d*_ and *K*_*d*_ were set in (10, 20) and (134, 235) respectively.

#### The correction of desired trajectory based on impedance controller

**Figure 9:**
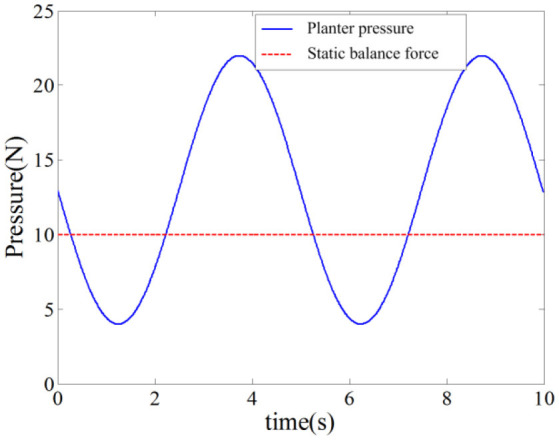
Plantar pressure and static balance force.

**Figure 10:**
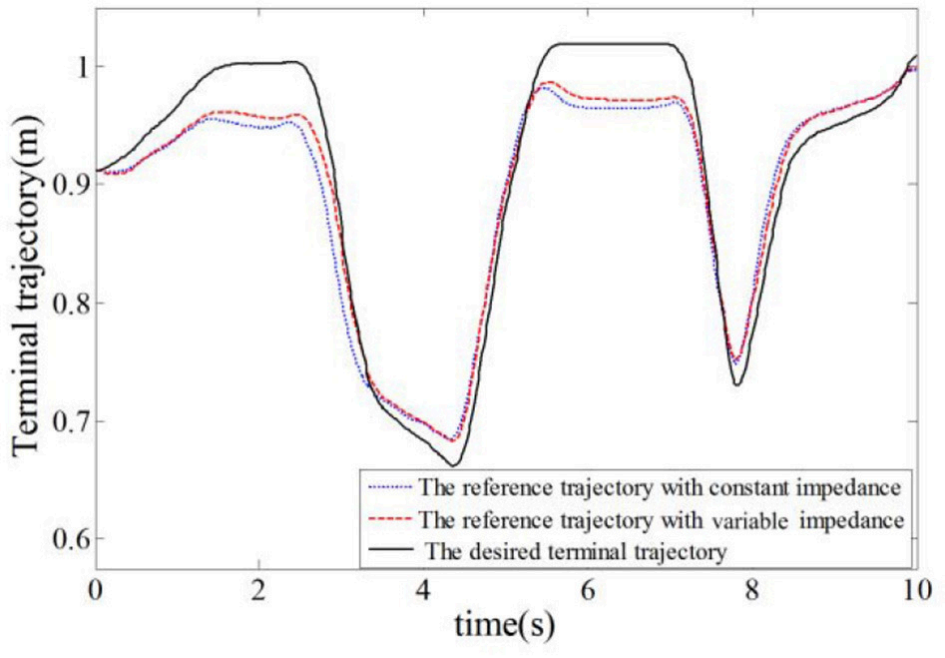
Trajectory of the rehabilitation robot.

In this simulation experiment, the plantar pressure was set as ***F***_int_ = 9*sin(2π*f***·***t*)+13, where *f* = 1.26, and the static balance force is 10 N. As shown in Figure 9, the plantar pressure is less than the static balance force over 0~2 s and the plantar pressure is greater than the static balance force over 2~5 s. To verify the validity of the reference trajectory corrected by variable impedance controller and compare it with the constant impedance controller, the impedance coefficients were set as *K* = 220, *B* = 14, as shown in Figure 10. From 0.8 to 2.5 s, the subject's lower limb is in the transition state from extension to flexion, the plantar pressure is less than the static balance force, and the value of reference trajectory is less than the desired trajectory. From 3.5 to 4.5 s, the subject's lower limb is in the transition state from flexion to extension, the plantar pressure is more than the static balance force, and the value of the reference trajectory is higher than the desired trajectory. Combining Figures 8, 10, we can find that from 1.5 to 2.5 s, the lower limb activity is significantly enhanced, the stiffness coefficient is more than 220, the damping coefficient is more than 14, and the reference trajectory modified by variable impedance controller is more closer to the desired trajectory compared with that of the constant coefficients impedance control. In other words, subjects are encouraged to perform a flex movement. From 2.5 to 5 s, the subject's lower limb activity decreased and the stiffness and damping coefficients became smaller. The deviation of trajectory correction is increased, which indicates the compliance performance of the rehabilitation robot system, and provides rehabilitation assistance that matches the subject's motion ability.

#### The reference trajectory tracking of rehabilitation robot

**Figure 11:**
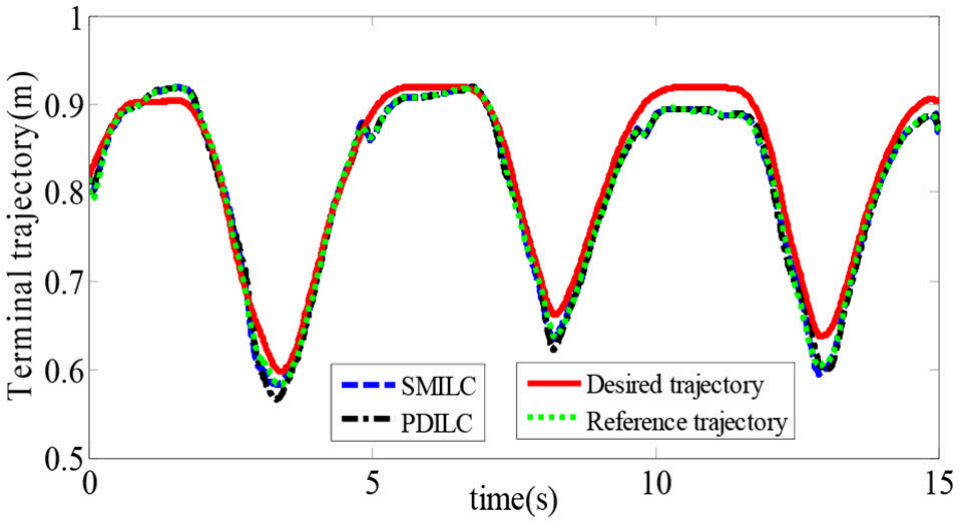
Tracking trajectory of the rehabilitation robot.

**Figure 12:**
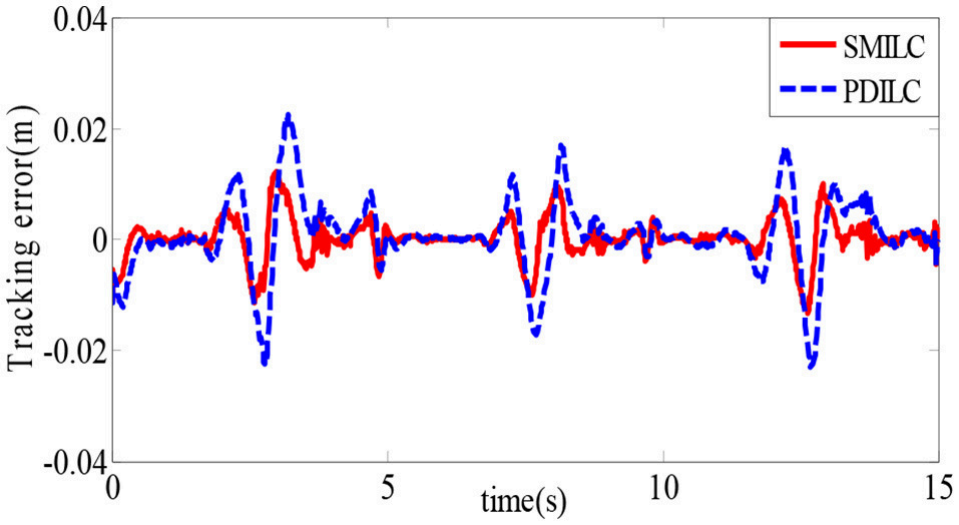
Tracking errors of the rehabilitation robot.

To verify the effectiveness of the sliding mode iterative learning control based on the variable boundary saturation function, we designed a controller to realize the terminal trajectory tracking of the lower limb rehabilitation robot and compared it with the PD iterative learning control algorithm (PDILC). In this paper, the SMILC algorithm parameters are set as *c*_1_ = *c*_2_ = 50, ${\bar{\tau }}_{U}={\left[\begin{array}{cc} 2 & 2 \end{array}\right]}^{T}$, ${\bar{\tau }}_{L}={\left[\begin{array}{cc} -2 & -2 \end{array}\right]}^{T}$, *p* = 1, *q* = 3, **ε** = [0.5 0.5]^*T*^, and *k* = 10 and the number of iterations is *i* = 15; the PDILC algorithm parameters are respectively set as $kp={\left[\begin{array}{cc} 50 & 0\\ 0 & 50 \end{array}\right]}^{T},\;kp={\left[\begin{array}{cc} 50 & 0\\ 0 & 50 \end{array}\right]}^{T}$, and the number of iterations is set as 15. The tracking trajectory obtained by SMILC and PDILC algorithms are shown in Figure 11 and the tracking errors of the algorithms are shown in Figure 12.

As shown in Figure 11, with the change of the plantar pressure and impedance coefficients, the controller can adaptively correct the desired trajectory to obtain the reference trajectory, and both the SMILC and PDILC algorithms can achieve stable terminal trajectory tracking of the lower limb rehabilitation robot. However, the SMILC algorithm tracking error is kept within ±0.013 m and the convergence time is 0.33 s, while the tracking error of PDILC algorithm is ±0.025 m and its convergence time is 0.52 s, as shown in Figure 12, which indicate that the SMILC algorithm proposed in this paper can track the terminal trajectory with less time and smaller errors. Three abnormal jitters can be seen in the trajectory tracking process, which are related to the lower limb transition state from flexion to extension.

#### The statistical analysis of the trajectory tracking error

**Figure 13:**
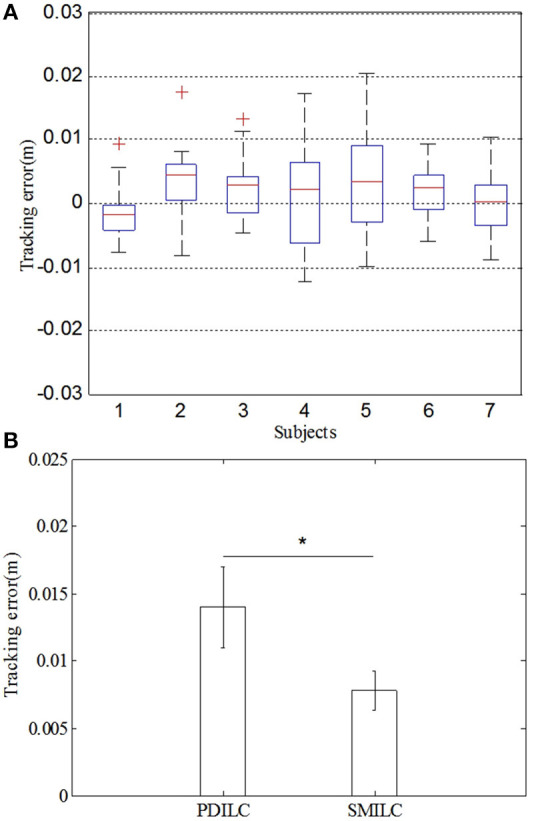
The statistic result of tracking errors for PDILC and SMILC. **(A)** The tracking error of 7 subjects for SMILC. **(B)** Comparison of tracking error between PDILC and SMILC.

To further validate the feasibility and effectiveness of SMILC, we made a statistical analysis of the trajectory tracking error of PDILC and SMILC. The statistic result of tracking errors were shown in Figure 13. In Figure 13A, the statistic of tracking error of SMILC was performed, which came from the 7 subjects' lower limb training with rehabilitation robot. Each subject's tracking trajectory was repeated 10 times with SMILC. As it can be seen, all of 7 subject's tracking errors [*F*_(6, 3)_ = 1.49, *p* = 0.191] vary up or down at zero and have little significant difference each other, which means that based on the proposed SMILC algorithms, the terminal trajectory tracking can be realized with little error for different subjects. In Figure 13B, taking subject S2 as an example, the mean and variance of the absolute value of the tracking errors were calculated separately for PDILC and SMILC. As it can be seen, there is significant difference between PDILC and SMILC [*F*_(1, 18)_ = 13.71, *p* = 0.000], which is represented by “*”, as shown in Figure 13B, and the mean and variance of the absolute value of the tracking errors for PDILC are obviously bigger than that for SMILC, which means that more stable trajectory tracking is realized based on the SMILC.

## Conclusion

In this paper, we proposed an advanced adaptive control method, which was devised with a dual closed loop control strategy based on the sEMG and plantar pressure. The variable impedance controller was designed to obtain the reference trajectory of the rehabilitation robot, making the reference trajectory more closer to the desired trajectory of patients. And the sliding model iterative learning control was designed with the variable boundary saturation function to track the terminal trajectory of rehabilitation robot. The results showed that the proposed control strategy could adjust the reference trajectory according to the motion intention of subject and realize the trajectory tracking more effectively. The advanced adaptive control method can improve the performance of the human-robot interaction and the robustness of the control system for lower limb rehabilitation robot. In addition, the proposed strategy could also be applied in the upper limb rehabilitation robots and others. Our future work will focus on the application of the proposed adaptive control method to the rehabilitation robot for patients.

## Author contributions

YD proposed the ideas of paper and wrote the contents. HW experimented and analyzed the experiment data of subjects. SQ and WY provided the results analysis. PX and XC provided the suggestions of paper.

### Conflict of interest statement

The authors declare that the research was conducted in the absence of any commercial or financial relationships that could be construed as a potential conflict of interest.
